# Low Yield of Genetic Testing in Serrated Polyposis Syndrome

**DOI:** 10.14309/ctg.0000000000000923

**Published:** 2025-09-26

**Authors:** Ira Upadhye, Husam Al Maliki, Victoria Cuthill, Andrew Latchford, Kevin Monahan

**Affiliations:** 1 Department of Surgery and Cancer, Imperial College London, London, UK;; 2 Norfolk & Norwich University Hospitals, NHS Foundation Trust, Norwich, UK;; 3 Centre for Familial Intestinal Cancer, St Mark's Hospital, London, UK.

**Keywords:** serrated polyposis syndrome, serrated polyps, colorectal cancer

## Abstract

**INTRODUCTION:**

Serrated polyposis syndrome (SPS) is clinically defined by the presence of multiple serrated polyps in the colon and rectum, and is associated with increased colorectal cancer risk. SPS is the most prevalent polyposis condition; however, its genetic basis remains poorly characterized. The British Society of Gastroenterology recommends gene panel testing for all patients with SPS to rule out other polyposis conditions. The aim of this study was to evaluate the diagnostic yield of genetic testing in patients with SPS.

**METHODS:**

We conducted a retrospective, cross-sectional analysis using the Polyposis Registry from St. Mark's Hospital, London, a national referral center in the United Kingdom. Patients with SPS who underwent genetic testing between April 4, 2009 and February 9, 2024, and met the SPS WHO criteria were included. Genetic variants were identified from test reports, and clinical data were extracted from medical records.

**RESULTS:**

In total, 573 people with SPS were identified in our registry, of whom 258 underwent genetic testing. Of these, 119 underwent target gene testing and 139 underwent multigene panel testing. No pathogenic variants were detected through targeted genetic testing. On multigene panel testing, pathogenic germline variants were found in 4 patients (2.9%), including 3 with Lynch syndrome (2 with *PMS2*, one with *MSH2*) and one with an *RNF43* variant.

**DISCUSSION:**

Genetic testing demonstrated a low diagnostic yield in this SPS cohort, suggesting undefined genetic risk or involvement of other pathophysiological factors. Therefore, genetic testing seems to have limited utility in patients with SPS and may primarily identify those with an incidental diagnosis of Lynch syndrome.

## INTRODUCTION

Serrated polyposis syndrome (SPS) is a precancerous condition characterized by the presence of multiple serrated polyps (SP) throughout the colon and rectum (1). SPS is associated with a significant risk of progression to colorectal cancer (CRC), making early identification and management critical. According to the World Health Organization (WHO) guidelines SPS is clinically diagnosed when an individual's cumulative lifetime count of SP meets one of the following criteria: (i) ≥5 serrated lesions/polyps proximal to the rectum all being ≥5 mm in size, with ≥2 being ≥10 mm in size or (ii) >20 serrated lesions/polyps of any size distributed throughout the large bowel, with ≥5 being proximal to the rectum (1).

SPS is the most common polyposis syndrome, with a UK prevalence estimated between 0.34% and 0.66% (2,3). A monogenic cause for SPS has not been identified and the molecular basis for the condition remains largely unknown (1,4,5). *RNF43*, a tumor suppressor gene, has been implicated in a small number of SPS cases (5,6); however, findings have been inconsistent (7,8). Murphy *et al. *(9) found that 9.6% of patients with SPS harbored pathogenic variants in CRC-related genes such as *RFN43, MUTYH*, and *CHEK2*, whereas others reported no pathogenic variants in genes such as *PTEN*, *SMAD4*, and *BMPR1A* (10). The evidence of a strong familial component is weak, although there are some clues that there may be a familial component to SPS in some cases (11–13).

In 2018, the UK Cancer Genetics Group developed the R211 panel to standardize genetic testing for early-onset colorectal cancer and polyposis syndromes (14,15). This panel, which uses next-generation sequencing, provides an efficient, cost-effective approach to cover a wide range of genes associated with increased CRC risk (16). The British Society of Gastroenterology has outlined which patients with SPS should undergo genetic testing to rule out other polyposis syndromes, such as *MUTYH*-associated polyposis and Peutz-Jegher syndrome, as dual pathology can occur (2,17,18). However, the evidence base for this recommendation was identified as being weak (2). In November 2020, St. Mark's adopted the R211 panel for patients with SPS meeting the British Society of Gastroenterology criteria for genetic testing (2,14,15).

The lack of understanding surrounding the genetic factors underlying SPS limits the ability to tailor personalized surveillance strategies for CRC risk. As a result, current surveillance guidelines for SPS rely on limited evidence and use a one-size-fits-all approach, which may lead to unnecessary procedures (2). Given that colonoscopies are invasive and carry inherent risks, identifying genetic factors where present, informs management of an individual but also the wider family. The primary aim of this study was to evaluate the diagnostic yield of genetic testing in patients with SPS and assess whether current guidelines should be revised.

## METHODS

### Study population

**Figure 1.**
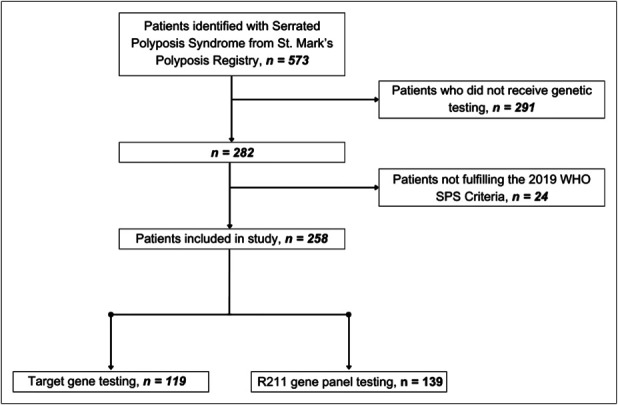
Flowchart of patients included and excluded in the study. SPS, serrated polyposis syndrome.

We conducted a cross-sectional review up to February 9, 2024 using prospectively collected electronic and paper records from the Polyposis Registry at St. Mark's Hospital, Harrow, United Kingdom. The purpose of this review was to evaluate the diagnostic yield of genetic testing in patients with SPS. The inclusion criteria for this study were patients who met the WHO diagnostic criteria for SPS and had undergone gene panel testing. In total, 258 patients with SPS were identified from April 4, 2009, to February 9, 2024 (Figure 1).

Patient data were extracted from the Polyposis Registry and electronic patient records. The following information was collected: patient demographics, colonoscopy and histology findings, and outcome of genetic testing. Colonoscopy and histology records were examined to determine which SPS criteria the patient fulfilled. Patient age was recorded based on the age at SPS diagnosis. Molecular results were obtained from genetic test reports stored in the Polyposis Registry and were updated if subsequently reclassified.

### Genetic testing and analysis

Between April 4, 2009 and November 3, 2020, targeted genetic testing was performed on 119 patients with SPS. During this period, testing was conducted on an individual, per-patient basis. The targeted genes included *GREM1* and *MUTYH*, with additional testing for *APC, POLE*, and *POLD1* performed in some cases. Consequently, some patients underwent testing for a single gene, whereas others were tested for multiple genes.

In November 2020, genetic testing transitioned to the R211 multigene panel test (MGPT) provided by the NHS Genomic Service (16). This panel includes genes associated with CRC and polyposis syndromes, specifically *APC, BMPR1A, EPCAM, MLH1, MSH2, MSH6, MUTYH, NTHL1, PMS2, POLD1, POLE, PTEN, RNF43, SMAD4,* and *STK11* (16). Between November 4, 2020 and February 9, 2024, 139 patients with SPS underwent R211 panel testing (Figure 1).

All genetic testing was performed in NHS-accredited laboratories using next-generation sequencing using the Illumina TruSight and equivalent platforms. Test results were interpreted based on the authorized report and updated if any variants were reclassified. Identified variants were classified as pathogenic, likely pathogenic (with >90% confidence in pathogenicity), or variants of uncertain significance (VUS).

The medical history of first-degree and second-degree relatives was recorded, where available, for individuals identified as having pathogenic variants, VUS, or carrier status in the targeted genes. CRC histories were extracted from medical records and findings related to SP were obtained from colonoscopy and histology reports.

### Statistical tests

In this study, descriptive statistics were used to summarize patient demographics and genetic testing outcomes. The Kolmogorov-Smirnov test indicated that the data were not normally distributed, so nonparametric statistical tests were applied. Nonparametric data, including the age of SPS onset, were presented as medians with interquartile ranges. Categorical variables (sex, age, smoking status, and WHO criteria) were reported as frequencies and percentages.

### Ethical approval

The research and development office at St. Mark's Hospital registered and approved the study as part of a service evaluation project. Therefore, this project did not require ethical approval, and data were anonymized.

## RESULTS

### Study population

In total, 258 patients met the study criteria (from a total cohort of 573 patients); the baseline demographics are provided in Table 1. The median age at SPS diagnosis was 42 ± 22.8 years (range 23–82 years). In addition, 136 patients (52.7%) were female, and 160 patients (62.0%) were White. According to the 2019 WHO criteria for SPS, 33 patients (12.8%) fulfilled WHO Criterion I, 135 patients (52.3%) fulfilled WHO Criterion II, and 90 patients (34.9%) fulfilled both WHO Criterion I and II. The most common clinical presentation for patients who were diagnosed with SPS was symptomatic (52.3%), which included rectal bleeding, change in bowel habits, and abdominal pain. Nine patients (3.5%) were referred because of a relative's history of SPS, and 20 patients (7.8%) were referred because of a relative's history of CRC. Where information regarding smoking status was available, over half of patients (54.6%) were smokers. Before SPS diagnosis, 3 patients were identified with Lynch syndrome. No other patient had a known hereditary CRC-related condition. Approximately, a quarter of patients (24.4%) had a personal history of CRC. In addition, where data were available, we did not observe a significant number of extracolonic cancers in our cohort.

**Table 1.** Characteristics of the study population

Characteristics	Total n = 258 (%)	Target gene n = 119	R211 panel n = 139
Sex n, (%)			
Female	136 (52.7)	60	76
Male	122 (47.3)	59	63
Ethnicity			
White	160 (62.0)	71	89
Asian	20 (7.8)	9	11
Black	7 (2.7)	4	3
Hispanic	3 (1.2)	2	1
Other	68 (26.4)	33	35
Unknown			
Age at diagnosis (yr)			
30 or less	15 (5.8)	11	4
31–40	97 (37.6)	45	52
41–50	52 (20.2)	10	42
51–60	38 (14.7)	20	18
61–70	36 (14.0)	20	16
71 or over	20 (7.8)	13	7
Smoker			
Never smoker	99 (38.4)	40	59
Ever smoker	119 (46.1)	48	71
Unknown	40 (15.5)	31	9
WHO criteria			
Criterion 1	33 (12.8)	15	18
Criterion 2	135 (52.3)	62	73
Criterion 1 and 2	90 (34.9)	42	48
Reason for referral			
Symptomatic^a^	135 (52.3)	60	75
Family history SPS	9 (3.5)	7	2
Family history of CRC	20 (7.8)	11	9
Incidental	13 (5.0)	6	7
BCSP^b^	41 (15.9)	20	21
CRC	40 (15.5)	15	25
CRC history			
Yes	63 (24.4)	33	30
No	195 (75.6)	86	109

BCSP, bowel cancer screening program; CRC, colorectal cancer; SPS, serrated polyposis syndrome; WHO, World Health Organization.

a Those who had symptoms, were referred for colonoscopy, and identified to have serrated polyps.

b Those identified from the NHS National Bowel Cancer Screening program.

### Genetic testing

There was a variation in the number of genes that were tested for in each patient. One hundred nineteen patients underwent targeted gene testing and 139 patients underwent the R211 MGPT (for 16 significant genes associated with CRC).

**Figure 2.**
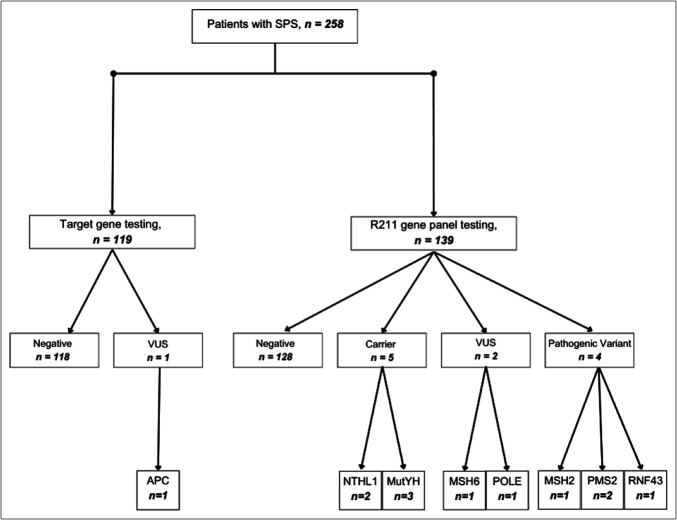
Flowchart showing the outcome of genetic testing in serrated polyposis syndrome cohort. VUS, variant of unknown significance.

Of the 119 patients who underwent target gene testing, one patient was identified with a VUS and the remaining 118 patients had no variant. Of the 139 patients who underwent MGPT testing, an actionable pathogenic variant was only found in 4 patients (2.9%). In addition, 5 patients (3.6%) were identified as heterozygous carriers and 2 patients (1.4%) were identified with a VUS. In the 128 patients who underwent R211 gene panel, no variant was found (Figure 2). When combining both cohorts, only 4 patients (1.6%) had an actionable pathogenic variant.

The number of patients tested for each gene is described in Table 2. *MUTYH* was tested the most frequently (n = 245), and *GREM1* was tested the least frequently (n = 70). In total, 2,431 genes were tested across 258 patients, identifying 12 germline variants (0.5%) (including pathogenic variants, VUS, and carriers).

**Table 2.** Number of patients tested for each gene and the result

Genes tested (n = 16)	No. of patients tested	Pathogenic variant	VUS	Heterozygote carrier^^a^^	Total germline variant, n (%)
APC	172	0	1	n/a	1 (0.6)
BMPR1A	139	0	0	n/a	0 (0.0)
EPCAM	139	0	0	n/a	0 (0.0)
GREM1	70	0	0	n/a	0 (0.0)
MLH1	139	0	0	n/a	0 (0.0)
MSH2	139	1	0	n/a	1 (0.7)
MSH6	139	0	1	n/a	1 (0.7)
MUTYH	245	0	0	3	3 (1.2)
NTHL1	139	0	0	2	2 (1.4)
PMS2	139	2	0	n/a	2 (1.4)
POLD1	154	0	0	n/a	0 (0.0)
POLE	154	0	1	n/a	1 (0.7)
PTEN	139	0	0	n/a	0 (0.0)
RNF43	139	1	0	n/a	1 (0.7)
SMAD4	139	0	0	n/a	0 (0.0)
STK11	139	0	0	n/a	0 (0.0)
Total	2,431	4	3	5	12 (0.5)

APC, adenomatous polyposis coli; BMPR1A, bone morphogenetic protein receptor, type 1A; EPCAM, epithelial cell adhesion molecule; GREM1, Gremlin 1; MLH1, MutL homolog 1; MSH2, MutS homolog 2; MSH6, MutS homolog 6; MUTYH, MutY homolog; NTHL1, endonuclease III-like protein 1; PMS2, post-meiotic segregation increased 2; POLD1, DNA polymerase delta 1; POLE, DNA polymerase epsilon; PTEN, phosphatase and TENsin; RNF43, ring finger protein 43; SMAD4, mothers against decapentaplegic homolog 4; STK11, serine/threonine kinase 11; VUS, variant of unknown significance.

a Heterozygote carriers are only relevant for recessively inherited genes *MUTYH* and *NTHL1* (30).

### Genetic variants

The characteristics of patients identified with a genetic variant are provided in Table 3. In total, 4 patients had pathogenic variants: one with *RNF43*, one with *MSH2,* and 2 with *PMS2*; the latter 3 had Lynch syndrome. The father of the patient with an *RNF43* variant had SP and a history of CRC. Colonoscopy data demonstrated that he did not fulfil the 2019 SPS WHO criteria. He was not tested for *RNF43*. The son of the patient with an *MSH2* variant has SPS and is also a patient in our study cohort. He received R211 gene panel testing, which identified no pathogenic variants. The patient with *PMS2* exon 1 deletion has a history of sigmoid cancer (Table 3).

**Table 3.** Details of genetic variants identified and characteristics of patient and family

Gene affected	Genetic variant	WHO criterion	Age at SPS diagnosis	Hereditary CRC conditions	History of CRC (age)	Personal cancer history	Family history of SPS (age)	Family history of CRC (age)
Pathogenic variant								
*RNF43*	c.471del p.(Thr158ProfsTer6)	N/A	47	N	N	N	N	Father (67); Paternal grandmother (7)
*MSH2*	c.1407deIT p.Val470X	II	69	Lynch syndrome	N	Endometrial (54)	Son (41)	Father (71); Paternal uncle (58); Paternal grandmother (61)
*PMS2*	c.137G>T p.(Ser46Ile)	II	38	Lynch syndrome	N	N	N	N
*PMS2*	Exon 1 deletion	I and II	73	Lynch syndrome	Y (39)	N	N	Mother (60)
Variant of unknown significance								
*MSH6*	c.1450G>C p(Glu484 Gl)	I and II	67	Lynch syndrome	Y	N	N	Mother (90)
*POLE*	c.1337 G>A p.Arg446Gln in exon13	II	24	N	N	N	N	N
*APC*	c.[7472T>C] p.Met2491Thr	II	37	N	Y	N	N	N
Heterozygous carrier								
*MUTYH*	c.821G>A; p.(Arg274Gln)	I	82	N	Y (81)	N	N	N
*MUTYH*	c.536A>G; p. (Tyr179Cys)	II	58	N	Y (56)	N	N	N
*MUTYH*	c.1187G>A; p.(Gly396Asp)	II	66	N	Y (39)	N	N	N
*NTHL1*	c.98G>A; p.(Arg33Lys)	II	32	N	Y (32)	N	N	N
*NTHL1*	c.244C>T; p.(Gln82)	I and II	39	N	N	N	N	Maternal cousin (40)

Age reported in years.

CRC, colorectal cancer; SPS, serrated polyposis syndrome.

## DISCUSSION

This retrospective, cross-sectional analysis conducted at a specialist tertiary center, contributes to the limited evidence surrounding germline variants associated with SPS. Gene panel testing identified a pathogenic variant in only 1.5% of patients, aligning with similar studies (9,10). In addition, a variant detected in *MSH6* *(c.1450G>C)* has been classified as a VUS; however, Clinvar reports conflicting classifications of pathogenicity; if pathogenic, it may slightly alter the overall diagnostic yield (19). Arguably this emphasizes the point that the main benefit of diagnostic testing may be to identify coexisting diagnoses, such as Lynch syndrome and SPS, which may improve precision in care.

This is the first study to use the R211 gene panel in patients with SPS, and we found a pathogenic variant in *RNF43* (Table 3). This matched the variant identified by Murphy *et al.* (9) but differed from the splice site variant identified by Yan *et al.* (5). In addition, familial involvement was observed, consistent with Yan *et al. *(5), who reported the same *RNF43* variant in four family members. However, other multicenter studies have found no *RNF43* variants in SPS cases (9,10) reaffirming its rarity.

All *MUTYH* carriers in our study had CRC, which is consistent with historical reports that *MUTYH* carriers have a two-fold increased CRC risk (20). However, routine testing for monoallelic carriers is not recommended for SPS cases given the high prevalence of monoallelic *MUTYH* carriers in white populations (20).

Interestingly, we identified a dual pathology between SPS and Lynch syndrome in 3 patients, suggesting the serrated pathway may contribute to Lynch syndrome CRCs (21,22). This dual pathology has been previously reported but may be over-represented because of ascertainment bias (23). Future investigations are required to identify whether dual pathology has an additive or cumulative cancer risk.

The most recent SPS study on genetic factors divided its cohort into 2 subgroups: those with most SP in the proximal colon and those with most in the distal colon (24). Pathogenic variants were identified only in the proximal colon subgroup, which may be attributed to the differences in the embryonic origins of the proximal and distal colon (25). This finding could prompt future SPS research to focus on polyp location when investigating genetic involvement.

Our SPS cohort showed no gender predominance, similar to a recent meta-analysis (26), and a median diagnosis age of 42 years, which is lower than previous reports (27). This younger age may be attributed to our use of family registry data, whereas other studies relied on hospital data. The predominance of White patients in our cohort aligns with previous studies indicating a higher prevalence of SPS in Whites (28,29). Finally, a fifth of our cohort had CRC, consistent with the estimated 15% and 29% risk in other studies (23,26).

NHS-funded constitutional genetic testing has traditionally only been offered to individuals with a likelihood of at least 10% for identifying a causative, clinically-actionable, constitutional pathogenic variant (30). The data presented in this study fall significantly below this threshold, raising questions about the value of genetic testing in SPS. These findings suggest that current guidelines for such genetic testing should be re-evaluated.

Several limitations must be considered. The complete R211 gene panel was assessed in only half of our cohort, potentially underestimating findings. The evolution of target test regions and sensitivity also introduces time-varying confounding throughout the study period, especially since 3 patients had a VUS. Data from the Polyposis Registry introduced ascertainment bias, as referrals may not represent all SPS cases. Detection bias could have occurred because of varying interpretations of colonoscopies and pathology findings by different specialists, particularly data dated back to 1990. In addition, smoking, alcohol use, and BMI data were incomplete, limiting the ability to assess confounding variables which may influence the pattern of inheritance of SPS (31). Finally, this study focuses on UK-based clinical practice and guidelines, which may limit the generalizability of our findings to international settings where recommendations for germline testing in SPS differ. Notably, some guidelines, such as those from the NCCN, currently recommend genetic testing only in the presence of additional clinical features (32).

The low diagnostic yield of pathogenic variants in patients with SPS raises questions about current guidelines and highlights the need to explore alternative pathways for SP development in future research. Multicenter studies on an international scale are essential. Expanding the gene pool and assessing family history through international collaboration will yield more comprehensive data, leading to a better understanding of the condition's genetic etiology. Longer follow-up studies are needed to assess CRC risk more accurately, ideally from 10 to 15 years, as this is the average time taken for a serrated polyp to become cancerous (33). This will help identify which individuals may require more frequent colonoscopy screenings. Finally, a national SPS registry, similar to the Lynch Syndrome Registry, could be introduced to improve patient identification and management (34).

The study highlights the low diagnostic yield of pathogenic variants in SPS, suggesting other factors contribute to its development and that current guidelines should be revised. Future research should focus on understanding the pathophysiology behind SP development, establishing family databases, and developing tailored surveillance strategies for SPS.

## CONFLICTS OF INTEREST

**Guarantor of the article:** Kevin Monahan, PhD.

**Specific author contributions:** V.C., and K.M.: study design and methodology. I.U.: data collection. I.U., and K.M.: analysis. I.U., H.A., A.L., and K.M.: interpretation of the results. I.U., H.A., and K.M: drafting the manuscript. All authors: reviewing, editing the manuscript and approving the final draft.

**Financial support:** None to report.

**Potential competing interests:** None to report.

**Study Highlights**

**WHAT IS KNOWN**

✓ Serrated polyposis syndrome (SPS) is associated with increased colorectal cancer risk.
✓ The British Society of Gastroenterology and UK National Genomic Testing Directory recommends genetic testing in patients with SPS to rule out other polyposis conditions, but guideline recommendations are based on limited and low-quality evidence

**WHAT IS NEW HERE**

✓ Two hundred fifty-eight patients with SPS who underwent genetic testing at the St. Mark's Hospital Polyposis Registry were analyzed.
✓ Our study showed a low diagnostic yield of 1.5% for actionable pathogenic variants, suggesting an undefined genetic basis or alternative pathophysiological mechanisms in patients with SPS. This yield was lowest when a selective approach was taken to genetic testing, compared with multigene panel testing.
✓ Pathogenic variants in *RNF43*, a tumor suppressor gene, was identified in one patient with SPS highlighting its known potential role but in a small number of SPS cases
✓ Findings highlight the potential overuse of genetic testing under current guidelines and call for targeted, evidence-based revisions to optimize resource use and patient care.

## ABBREVIATIONS:

CRC: colorectal cancer
MGPT: multigene panel testing
SP: serrated polyps
SPS: serrated polyposis syndrome
VUS: variant of unknown significance
WHO: World Health Organization
